# Impact of Exercise on Fatigue in Patients Undergoing Dialysis in a Tertiary Care Hospital

**DOI:** 10.7759/cureus.35004

**Published:** 2023-02-15

**Authors:** Vijay Samuel Raj V, Prashanth V Mangalvedhe, Manjunath S Shetty, Deeya C Balakrishnan

**Affiliations:** 1 Department of Sports Sciences/Physiotherapy, JSS College of Physiotherapy, Mysuru, IND; 2 Department of Physiotherapy, School of Allied Health Sciences, REVA University, Bengaluru, IND; 3 Department of Nephrology, JSS Medical College and Hospital, JSS Academy of Higher Education and Research, Mysuru, IND; 4 Department of Physiotherapy, JSS Hospital, Mysuru, IND

**Keywords:** chronic kidney disease, aerobic exercise, fatigue, physical activity, exercise, hemodialysis, chronic kidney disease (ckd)

## Abstract

Background

Chronic kidney disease (CKD) is a current public health problem associated with progression to end-stage renal disease (ESRD), cardiovascular disease, and increased mortality rates. The disease is progressive. It is estimated that there are about 20-25 patients with milder kidney damage for every patient on renal replacement therapy. Physical activity is one of the critical elements for the prevention of chronic diseases and exercises in CKD help to improve muscular strength, cardiorespiratory endurance, overall function, and quality of life. Fatigue can cause an inability to perform exercises and can affect physiological and psychological function. There is a need to analyze the effects of exercises on fatigue in outpatients undergoing dialysis in the Indian rural population.

Methods

This study was a randomized, controlled, interventional, single-center trial. The participants diagnosed with ESRD and who were on hemodialysis were randomly assigned to group A, the exercise group (EG), which had exercise training during dialysis, and group B, the control group (CG), which had no added exercises during dialysis or at home and followed a standard routine method. The outcome measure of fatigue was evaluated through the total Fatigue Assessment Scale (FAS) score at 0, 12, 24, and 36 weeks. The exercise was pilot tested and planned based on the guidelines and carried out during hemodialysis two days a week. It was followed up with a five-day home exercise program when the patients did not undergo dialysis.

Results

A total of 48 participants were chosen for the study, of which 30 participants completed 36 weeks of training, with exercise adherence of more than 60%. There was a statistically significant difference in FAS between the two groups (F (3, 84) = 10.513, P < 0.001) at a 95% confidence interval (P < 0.05). Post hoc comparisons between groups at baseline and at 12 weeks indicated that there was no significant difference in FAS (P = 0.271 and P = 0.08), but recorded a significant difference (P = 0.001) at 24 and 36 weeks, respectively, between the EG and CG.

Conclusions

The results indicate that the intradialytic exercise intervention was effective in reducing the level of fatigue in outpatients undergoing dialysis on a long-term exercise program.

## Introduction

Chronic kidney disease (CKD) is a debilitating disease and can progress to end-stage renal disease (ESRD), also called kidney failure, requiring dialysis or renal transplantation. ESRD is defined as a glomerular filtration rate (GFR) of less than 15 mL/min and is characterized by a loss of function gradually over time, which is indicated by a reduction in GFR and the presence of albuminuria. There is an increase in the life expectancy of Indians to 66 years reported as of 2013 and the prevalence of lifestyle diseases is also rising in India [1]. Diabetes and hypertension account for 40-60% of cases of CKD [1]. It has been estimated that the age-related incidence rate of ESRD is 229 per million in India and as there is a lack of community screening programs, it may result in the late detection of patients in the advanced stage of the disease [2].

Patients undergoing hemodialysis (HD) may suffer side effects such as hypotension, muscle cramps, excessive fatigue, inability to do work, and reduced physical activity. Hypotension can be caused by the drop in fluid levels during dialysis and can cause dizziness. It varies from short-term complications to long-term complications. Asymptomatic reduction in blood pressure (BP) during or immediately after dialysis is seen to occur in approximately 20-30% of people as a result of inadequate cardiovascular response when a large volume of water is removed during a short period of time [3]. Painful muscle cramps, usually in the lower extremities, are also common in patients undergoing HD, mainly at the end of each session. This increased incidence has been shown due to varying magnesium concentrations in the dialysate [4,5]. Fatigue, where the patients feel exhausted, is a common side effect of long-term dialysis. It is due to the following reasons: dietary restrictions, overall stress, and the impact of dialysis on the body. There may be increased muscle catabolism in dialysis patients caused due to insulin resistance, inflammation, or acidosis, which may lead to muscular fatigue and physical inactivity [6]. These patients also feel insomnia and bone and joint pain because of renal osteodystrophy, which makes bones thin and weak, and CKD may also weaken the muscles [7]. Studies have confirmed that exercise training in patients with CKD results in increased muscle mass and improvements in oxidative metabolism. Exercises are mentally and physically beneficial in patients undergoing HD, which may maintain the strength and integrity of muscles and can improve kidney function [8].

By performing exercises, they can carry out their daily activities, such as household work, and stay as independent as possible. In the Indian population, the complications remain the same, with an increasing incidence rate and dependency because of a lack of awareness about the effects of exercise on dialysis patients. Despite the benefits of exercise, patients are not exposed to the exercise regime during HD or at home. The prescription of training for CKD patients is lesser than usual compared with prescribed exercise for other chronic diseases. Exercises are noteworthy, considering that physical activity levels among CKD patients are significantly lower than healthy individuals [9]. Moreover, low aerobic capacity, a biological fitness marker that can be improved by exercise, has been pointed out to be the strongest predictor of mortality among ESRD patients. Patients with CKD should be given more importance in supervision and prescribing exercise programs [10]. Adherence to exercises is an essential prerequisite for successful exercise programs for musculoskeletal disorders [11].

Thus, exercise promotes functional ability, decreases fatigue, and may improve the quality of life. As the benefits of exercise could also apply to CKD patients, physical activity should be considered a significant component of treatment in all stages of the disease [12]. An exercise program that includes a supervised and home-based training phase is effective and safe in patients with CKD [13]. There is a need to analyze the effects of exercises on fatigue in outpatients undergoing dialysis in the Indian rural population.

This paper was presented as a poster at the Annual Meeting of the Korean Society of Nephrology (KSN) 2019 in Seoul, South Korea. The paper has attracted interest in terms of the importance of exercise during dialysis and has been an integral part of the rehabilitation of patients undergoing HD in the hospital.

## Materials and methods

This study was a randomized, controlled, interventional, single-center trial, with the allocator and provider blinded. By block randomization, the participants were randomly allocated into groups A (exercise) and B (control) using chits, where the allocator was blinded. Exercise training for ESRD patients was provided during HD and as a home exercise program (HEP) for group A and standard routine methods with no specific planned exercise program were followed for group B (control). The control group performed only hand grip exercises as followed during the routine HD. The study was approved by the Institutional Ethical Committee of JSS Medical College, JSS Academy of Higher Education and Research, formerly known as JSS University (approval number: 29/2007/2017-18).

The study population was patients diagnosed with ESRD with GFR less than 15 mL/min and undergoing dialysis as outpatients. Participants who could walk and be independent in function and carry out basic activities of daily living were included in the study. The screening of the participants for the inclusion criteria and for eligibility to perform exercises was carried out by the consulting nephrologist and physician, who ruled out the contraindications through a checklist and physical examination. Patients with recent fractures of extremities, neurologically unstable, psychologically unstable, and cognitive impairment were excluded. The purpose and benefits of the study were explained to the participants, and their informed consent was obtained.

The exercise protocol was designed taking reference from the Kidney Health Australia guidelines [14], as there was no published evidence on exercise protocol tested on the Indian population. The intradialytic exercise (IDE) protocol was tested and validated by a pilot study conducted in the same setting prior to the recruitment. The feasibility and applicability of the exercises were thoroughly assessed and modified according to the department's usual care and recent evidence. The activities included in the intervention program were warm-up, stretching, and strengthening exercises for the non-fistula limb and lower limb musculature using resistant bands. The protocol consists of three components: flexibility, dynamic strength, and endurance. The exercises were tailor-made based on frequencies, intensity, and duration and were calculated based on the Karvonen method. The maximum heart rate (MHR) was subtracted by resting heart rate (RHR) and multiplied by the percentage of maximum intensity of exercise and added with the RHR (Karvonen = (MHR - RHR) x percentage of maximum intensity + RHR). The maximum repetition rate (MRR) was calculated by instructing the patient to complete the maximum number of repetitions using the resistance of his/her comfort till the fatigue and/or pain or discomfort stops to continue. The number of repetitions was recorded at the baseline and during each of the sessions. MRR of 50-70% was considered during the first 16 weeks and gradually incremented to 5%. The strength was assessed using the resistance bands of different colors using MRR. The flexibility included stretching and active range of motion (RoM) exercises. The dynamic strengthening was achieved using the resistance band and the endurance training was done using a resistance adjustable cycle ergometer (Pedo Cycle, Fastcure, Bengaluru, India). All exercises were carried out during HD (Table 1).

**Table 1:** Exercise program standardization for CKD patients undergoing hemodialysis § Resistance is measured using resistance bands using resistance in pounds (lbs.) and maximum repetition rate (MRR). # Endurance is measured using resting heart rate (RHR) and maximum heart rate (MHR). ** Flexibility is measured by the joint range of motion (JROM) using a standard goniometer.

Duration	Dynamic resistance training^§^	Endurance training^#^	Flexibility**
	Using resistance band exercises	Cycle ergometer	Self-stretching exercises
Baseline test	Maximum repetition rate (MRR): 1 min	Resting heart rate (RHR), maximum heart rate (MHR)	JROM – Goniometer for shoulder/elbow/wrist/hip/knee/ankle
Month 1	50% of MRR	0.5x (MHR - RHR) + RHR	5 rep x 1 set for upper limb, lower limb (dialysis limb is excluded). Warm up = 5 minutes; followed by stretches
Month 2	65% of MRR	0.65x (MHR - RHR) + RHR	
Months 3 & 4	70% of MRR	0.75x (MHR - RHR) + RHR	
Month 5	Test for MRR	Test RHR and MHR	
Months 6-9	Each month increases by 5%		
Exercise parameter	1 set, two times/week, 30 seconds rest in between sets	Pedo Cycle – 5 to 20 minutes (or when Karvonen’s equation value is achieved)	Prior to resistance exercise/dialysis (HD). Active exercises to all joints (distal to proximal)

The exercise was terminated if the patient developed symptomatic pain or discomfort and changes in physiological function (heart rate (HR) > MHR with or without excess fatigue, BP > 180/110 mm Hg). The vitals were regularly monitored throughout the exercise program using a pulse oximeter and standard BP apparatus during HD, and fatigue was assessed subjectively. A structured HEP was prescribed and taught to the patients and attendees to follow up on the remaining five days of the week when they did not undergo dialysis. A printed HEP with an adherence sheet was given to all the participants enrolled in the experimental group. There were no changes to methods after trial commencement.

The baseline screening included all upper and lower limb joints' RoM. The RoM was assessed using a universal goniometer, measured in degrees, and was done by the same physiotherapist (investigator), who had expertise in the measurement tools and evaluation. The strength of elbow flexors and extensors, knee extensors and flexors, and hip abductors was checked by MRR using resistance bands (Active Band, Vissco Healthcare Pvt. Ltd., Mumbai, India) of either red, green, or blue color with a resistance of 3.5 lbs, 4.5 lbs, and 5.5 lbs, respectively. The range of the resistance bands (red, green, and blue) was used for this study, which was determined through the pilot study conducted on a similar population to standardize the methods and exercise protocol. The resistance was individualized and determined starting at 10% of the resistance and the patient's comfort to perform the elongation at least to 50% or till the end RoM. During the strength testing and training, the resistance band was held in hand for the upper limb and winded on the leg, where the participant performed the movement in the direction against the resistance of the band. Assessment of the level of fatigue was done using the Fatigue Assessment Scale (FAS). The FAS is a 10-item general fatigue questionnaire with five questions on physical and mental fatigue [15]. Scores are done from 1 to 5 with variations for questions 4 and 10. The total FAS score is calculated by adding the scores on all questions, ranging from 10 to 50. A total FAS score < 22 indicates no fatigue, and a score ≥ 22 indicates fatigue. The study was controlled through the patient's follow-up with the outpatient department attending the dialysis sessions. The referred cases for the study based on the inclusion criteria were collectively done by the nephrologists and the two primary investigators.

**Figure 1:**
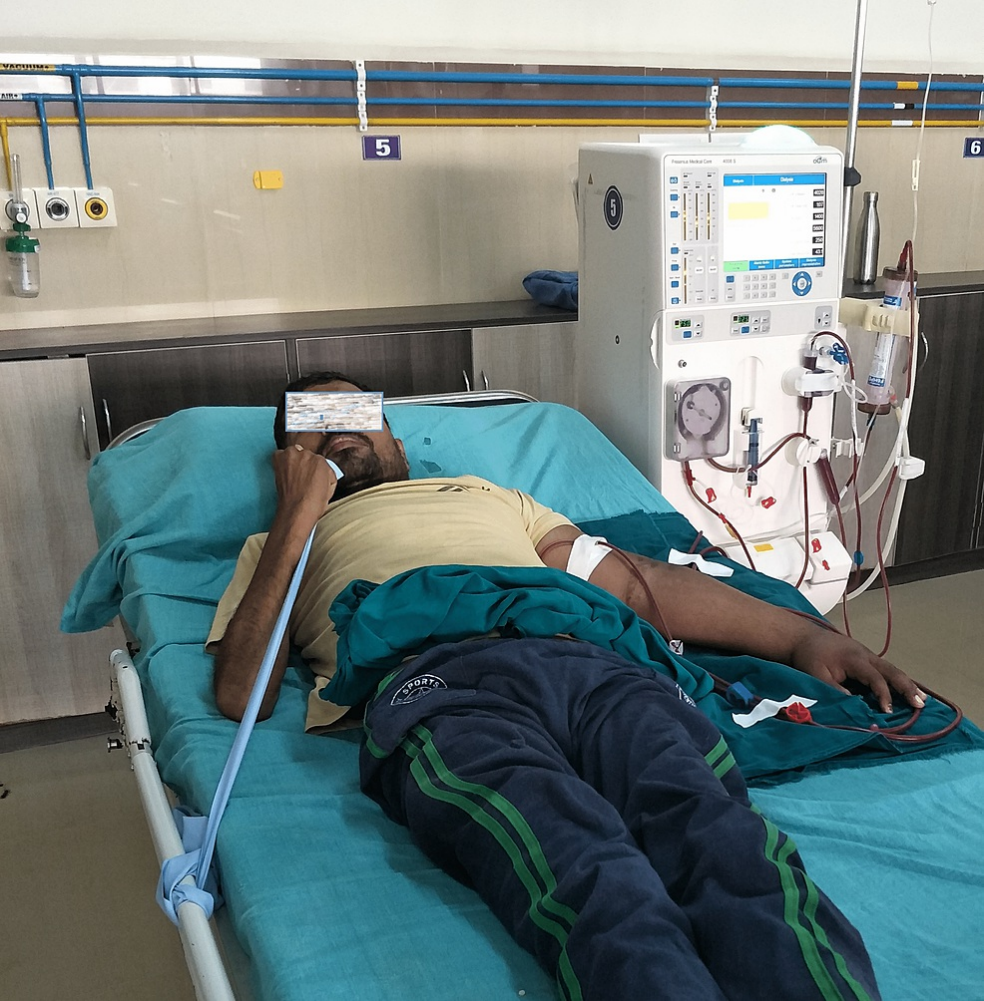
Strength training using resistance bands for the upper limb (elbow flexors)

The control group received no planned exercise program and underwent only dialysis. The experimental group received a strengthening, endurance, and flexibility program. The warm-up session included a brief general exercise per the participants' tolerance. It was followed by static self-stretching of all the joints of the upper and lower limbs (fistula limb excluded), which was incorporated at the beginning of the session. The strength training included the resistance exercise (Figure 1) with an appropriate color resistance band chosen to perform repetitions as tolerated initially and based on the MRR determined at the baseline.

**Figure 2:**
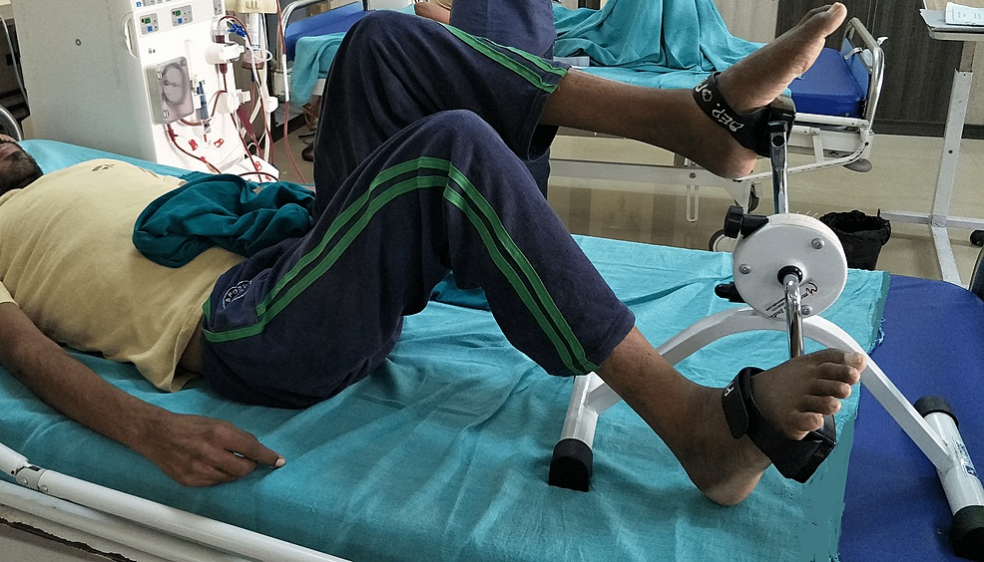
Endurance training using the Pedo Cycle during hemodialysis

The patients completed the activities twice a week during dialysis as outpatients in the hospital. Progression was done as per the exercise protocol. The endurance training was performed within the bed using a resistance adjustable cycle ergometer (Pedo Cycle) (Figure 2).

The exercise protocol (Table 1) was followed to achieve the goals, and patients were given 20 to 30 minutes of rest between the resistance and endurance exercises. Participants continued to reach the prescribed goal or until muscular fatigue, HR and/or BP variations, and or any new symptoms appeared. Qualified physiotherapists monitored the complete exercise intervention during the dialysis. At the end of 36 weeks, the MRR and FAS were re-assessed to know the muscle groups' strength improvements and fatigue levels. The exercises were planned with the reassessment of frequency, intensity, and duration for six to nine months, at the end of which the outcomes of FAS were taken. The scores of the FAS were assessed for normality using the Shapiro-Wilk test, and a normal distribution was observed. The data at each duration (0, 12, 24, and 36 weeks) were analyzed by repeated measures analysis of variance (ANOVA) at a 95% confidence interval (P < 0.05) using SPSS (version 22.0, IBM Corp., Armonk, NY).

## Results

**Figure 3:**
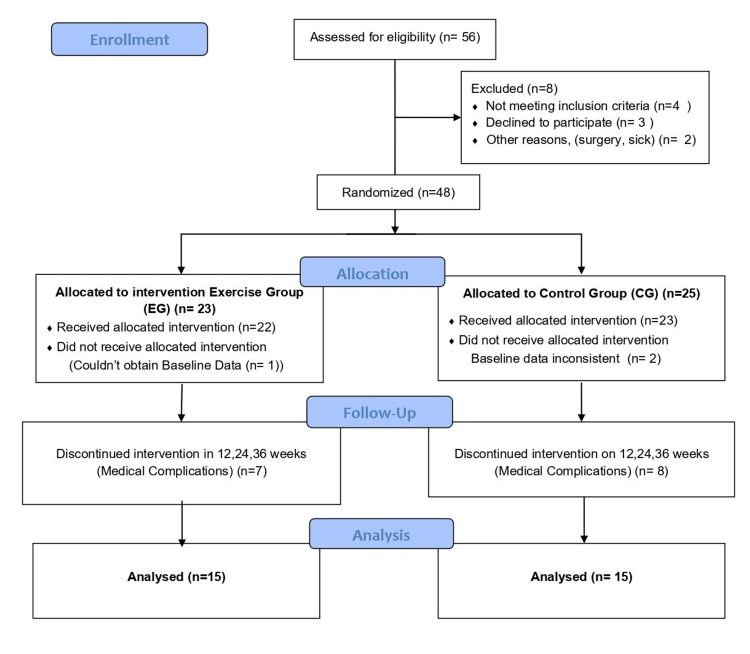
Flow diagram - participant flow through the study

A total of 48 participants fulfilling the inclusion criteria were enrolled in the study, out of which 42 were men. A total of 48 participants were randomly assigned to groups A and B: group A (the exercise group (EG)) consisting of 23 participants and group B (the control group (CG)) consisting of 25 participants. The study recorded a dropout rate of 42% (N = 18), eight in EG, and 10 in CG. Some reasons for dropping out were transfers, hospitalization, surgeries, and feeling sick. No dropouts were reported because of complications arising due to exercise during HD. The adherence to exercises recorded through the self-reported data chart was 52%. The other reasons recorded for the non-adherence to exercise at home and during dialysis were exhaustion, lack of interest, and motivation. A total of 30 participants who were consistent and completed the study with more than 60% adherence to exercise were included in the data analysis (Figure 3).

The mean age of the participants (N = 30) was 47.10 years (SD: ±12.97). Since the sample size was small, the normality of FAS was essential for choosing an appropriate statistical method. A Shapiro-Wilk test showed that the distribution of FAS scores did not show a significant departure from the normality (W (30) = 0.949, P = 0.158). The outcome measure of fatigue was evaluated through the total FAS score, with the baseline recording of 20.67 (SD: ±4.90) in the experiment group and 23.33 (SD: ± 7.78) in the control group, showing that both groups were closely related with a mean difference (MD) of 2.66 and both were in high average fatigue score level. Post-tests were conducted at 12, 24, and 36 weeks, and significant differences were noted in both groups.

The experimental group recorded a decrease in the total FAS score at the end of 36 weeks, with a pre-post MD of 5.8 FAS total score, showing the clinical significance of the minimal clinically important differences (MCID) greater than four points (MCID > 4). At the end of 36 weeks, the control group recorded a substantial fatigue score of 29.80 (SD: ±4.93). A repeated measures ANOVA was performed to compare the effect of IDE on FAS scores (Table 2). There was a statistically significant difference in FAS between the two groups (F (3, 84) = 10.513, P < 0.001).

**Table 2:** Repeated measures ANOVA - within-subjects effects for FAS for EG and CG ^a ^Statistically significant at 0.05. FAS: Fatigue Assessment Scale; EG: exercise group; CG: control group.

Source	Sum of squares	Degree of freedom	Mean square	F	Sig.^a^
FAS group	682.492	3	227.497	10.513	0.000
Error (change)	1817.667	84	21.639		

Independent samples t-tests were used to make post hoc comparisons between groups (Table 3). A first and second Independent samples t-test at baseline and at 12 weeks, respectively, indicated that there was no significant difference in FAS scores between the EG and CG (P = 0.271 and P = 0.08). A third and fourth Independent samples t-test at 24 and 36 weeks, respectively, indicated that there was a significant difference in the FAS score (P = 0.001).

**Table 3:** Mean difference between the EG and CG of FAS scores over 36 weeks ^b^ Mean between-group difference suggests the comparison of FAS. A negative value of the MD estimate represents an effect in favor of the experimental group. FAS: Fatigue Assessment Scale; EG: exercise group; CG: control group; MD: mean difference.

Time-point		Sample t-test for equality of means			
		t	df	Sig. (2-tailed)	MD (95% CI)^b^
Baseline		-1.122	28	0.271	-2.66667
12 weeks		-1.815	28	0.080	-4.00000
24 weeks		-3.736	28	0.001	-6.46667
36 weeks		-9.897	28	0.000	-14.93333

## Discussion

The present study aimed to determine the effects of exercise on fatigue. The exercises prescribed were tailor-made and were delivered during the dialysis with complete monitoring by a physiotherapist. Also, a home exercise program was taught, and the HEP was monitored through a self-reported adherence chart provided to the patient and the caretaker. Only the patients who fulfilled to complete more than 60% of HEP were included in the analysis. Fatigue is the most frequently described and globally recognized as a disabling symptom [15]; especially in CKD, fatigue is reported as one of the most common and debilitating symptoms [16]. Severe fatigue symptoms are reported in 25% of CKD patients, and approximately 70% report fatigue. This current study found that almost 80% of participants reported fatigue ranging from mild to moderate and had variations during the FAS recording at 12, 24, and 36 weeks. The within-group analysis for the exercise group showed a reduction in the FAS scores, depicting a decrease in fatigue over a long duration. There were no clinically significant differences between the IDE (EG) and control group at the end of 12 weeks period. There are no studies reported on IDE and FAS to support this, but a study conducted in India [16] evaluated the quality of life, peak oxygen consumption, and fatigue using the rate of perceived exertion (RPE) and showed a significant improvement after 12 weeks of IDE but had its limitations as it was single group study. The pathophysiology of fatigue in CKD is multifactorial. It probably includes decreased oxygen delivery and dependency on anaerobic metabolism, leading to lactic acidosis, which can lead to chronic fatigue, skeletal muscle atrophy, sarcopenia, and depression [17]. Physical activity and exercise have been shown to improve fatigue. Due to added cardiovascular profile and mental health benefits, the exercises are recommended as an effective intervention to improve fatigue and physical function [18]. Recent studies have evidence that regular exercise benefits the physiological and psychological aspects of patients with ESRD. However, ESRD patients have reported having a high non-adherence level to exercise during dialysis and self-exercises. Some studies have reported that this may be due to a need for more motivation among patients and attendees. This present study reported a drastic decline in adherence to the HEP and during dialysis at the later stage after 24 weeks, which may be contrary to the study by Williams et al., who concluded that adherence was higher in patients with encouraging support groups and when compared to the other reported studies [19]. Motivation and counseling would help in actively participating in exercise programs in ESRD. The present study incorporated the methods to encourage the participants to exercise. Still, the prediction of this was beyond the scope of this study. Despite the challenges of exercise prescription during the HD, the study had encouraging participation from the patients and patients’ attendees, which may be due to patient education on the benefits of exercise. Also, the other reasons for active involvement would be instructions and referrals stating the importance of consulting nephrologists and treating physiotherapists. The exercise protocol used combined aerobic and resistance exercises, which may be the reason for the reported benefits of fatigue.

Strengths

The pilot study conducted for a similar population in the same setting before the recruitment and implementation was a strength of the study, as this helped to understand the prescription of frequency, intensity, and duration of the exercises and also measure the proper use of technology and methods. Incorporating a standardized exercise protocol tailor-made for each patient was an added strength. Randomization helped to eliminate the bias in evaluation and allocation. The motivation during the exercise program was done throughout with monitoring by a physiotherapist and supported by the medical team.

Limitations

The sample size was achieved per the requirement. Still, the dropout rate was more significant after 12 and 24 weeks of the exercise program; the reasons due to exercise were not reported during the study and hence were not explored. Despite encouragement to perform activities during dialysis, the supervised training was for only two days, and supervision at home was limited to patient self-report. The presentation of strength and endurance was beyond the scope of the result analysis, despite the recorded data at various durations, but was used only to prescribe and calculate the dosage parameters of the exercise.

Future directions

Safety and prescription based on guidelines must be incorporated, and further research to explore this is needed. The delivery of home exercises and monitoring systems can be included to explore adherence and effectiveness, which can also be done through telerehabilitation.

Novelty/innovation

The exercise will help to carry out daily living activities and can decrease fatigue. Exercising while on dialysis can improve function and reduce the risk of complications. Although known to the practitioner and the patients, the effects of exercise are not regularly applied in hospitals and HD centers. This study aimed to utilize the effects of exercise in decreasing the level of fatigue, thereby improving quality of life. The results obtained in the study will be an indicator to plan appropriate exercise intervention regularly. They will help to have a standard protocol and thus can be followed more effectively.

## Conclusions

The EG and CG reported a moderate fatigue score, with high normal values at baseline. The EG showed consistent fatigue scores during the 12 and 24 weeks. Post 36 weeks, the EG reported a significant decrease in FAS scores when compared to the CG. The results indicate that the exercise intervention was effective in reducing the level of fatigue in outpatients undergoing dialysis on a long-term program, but had little or no effect in the short-term exercise intervention.
